# Challenging the control through a single-cell perspective on normal adjacent tissue in colorectal cancer

**DOI:** 10.1016/j.isci.2026.115671

**Published:** 2026-04-09

**Authors:** Patricia Raude, Onur Mert Batmaz, Subhiksha Meenakshi Sundaram, Christina Parpoulas, Xiaodong Wang, Joana Aggrey Fynn, Jana Koch, Dina Mönch, Thomas Mürdter, Marc H. Dahlke, Dominik Saul, Robyn Laura Kosinsky

**Affiliations:** 1 Robert Bosch Center for Tumor Diseases, Bosch Health Campus, Stuttgart 70376, Germany; 2 Dr. Margarete Fischer-Bosch Institute of Clinical Pharmacology, Bosch Health Campus, Stuttgart 70376, Germany; 3 University of Tübingen, Tübingen 72074, Germany; 4 German Cancer Consortium (DKTK), German Cancer Research Center (DKFZ), Tübingen 72074, Germany; 5 Department for Surgery, Bosch Health Campus, Stuttgart 70376, Germany; 6 Cluster of Excellence iFIT (EXC 2180) “Image-Guided and Functionally Instructed Tumor Therapies”, University of Tübingen, Tübingen, Germany

**Keywords:** cancer, omics

## Abstract

While normal adjacent tissue (NAT) is used as a standard control in cancer studies, NAT in close proximity to the tumor can display tumor-like molecular features, which are associated with a higher risk of local recurrence in colorectal cancer (CRC). To investigate the cellular composition and molecular features of NAT in detail, we analyzed single-cell RNA sequencing and spatial transcriptomics datasets of CRC tissue, corresponding NAT, and healthy colorectal biopsies. Our findings revealed significant differences in both cellular composition and gene expression profiles between healthy, NAT, and tumor tissues. We identified differentially expressed genes between healthy and normal adjacent tissue for each cell type, as well as tumor-associated copy number variations within NAT. Furthermore, we identified gene panels that effectively distinguish between healthy tissue and tissues exhibiting tumor-associated gene expression profiles, including NAT and tumor tissue. Finally, we developed a pharmacological strategy to reverse tumor-associated gene expression patterns *in vitro*.

## Highlights

• Healthy and NAT colon tissues are transcriptionally distinct at the single cell level
• Differences between healthy tissue and NAT occur across all major cell types
• NAT contains both healthy and tumor-like epithelial cells, creating a hybrid profile
• NAT displays tumor-associated features, including copy-number variations

## Graphical abstract

**Figure:**
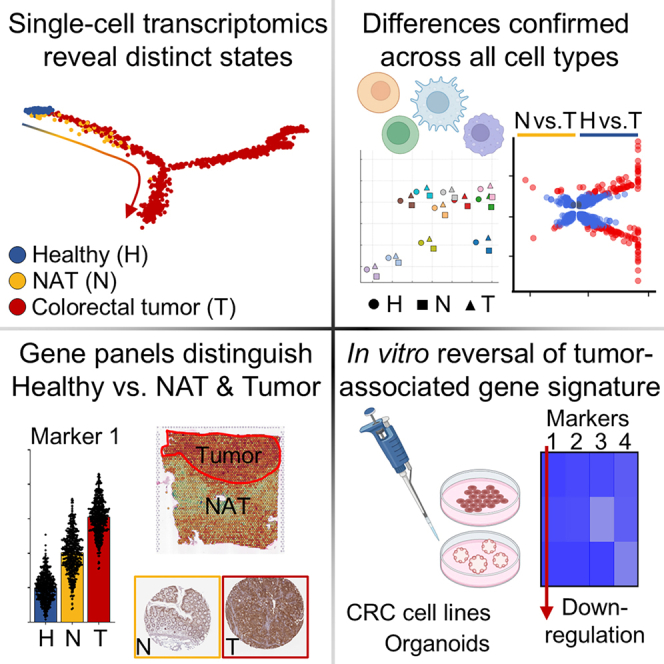

## Introduction

To identify molecular mechanisms underlying colorectal cancer (CRC) development and progression, tumor material is typically compared to normal adjacent tissue (NAT) upon surgery. Approximately 50%–70% of CRC patients undergo curative surgery.^1^ The resection includes proximal and distal margins of at least 2–5 cm to minimize the risk of local recurrence.^2^ In the clinical context, tissue identity is classified by a pathologist based on histological features.

Despite curative surgery, earlier studies described that approximately 10% of CRC patients develop local recurrence,^1^ suggesting that NAT may contain cancerous cells capable of forming new tumors. Indeed, while NAT displays histological normalcy, it was demonstrated that it does not show molecular normalcy.^3^ Previous studies have identified molecular differences, including allelic imbalance and altered telomere length in tissue adjacent to cancer lesions.^4^ Bulk RNA-seq analyses have revealed a panel of genes highly expressed in NAT but not in normal mucosa tissue, suggesting a relevant influence of tumors on the surrounding tissue.^5^ Further analyses have identified a NAT-specific transcriptomic signature of genes activated in tumor-adjacent tissue with an enrichment of extracellular matrix-associated genes only activated in NAT and likewise present in several cancer entities.^6^ These studies highlight the limitations of using NAT as a control tissue in gene expression studies of tumors.

While bulk RNA-seq provides valuable insights, it is limited in its ability to investigate the contributions and influences of specific cell types within the tissue. In contrast to bulk RNA-seq, single-cell RNA-seq (scRNA-seq) does not average out the transcriptomic signal from all analyzed cells, but measures the expression level of transcripts within each individual cell. This enables the analysis of the composition of a sample as well as examination of inter- and intracellular communication within the tissue sample, revealing biological heterogeneity.^7^^,^^8^

In this project, we demonstrate that the single-cell transcriptome of NAT is not comparable to healthy tissue across cell populations. In addition, the cellular composition of NAT samples differed from healthy as well as tumor tissue. We identified gene signatures that differentiate between healthy tissue and tissues exhibiting molecular features associated with tumorigenesis, including NAT, and used these to develop a pharmacological strategy in order to reverse the expression pattern of tumor-associated genes.

## Results

### Healthy and normal adjacent colon tissues are highly distinct at the single cell level

**Figure 1:**
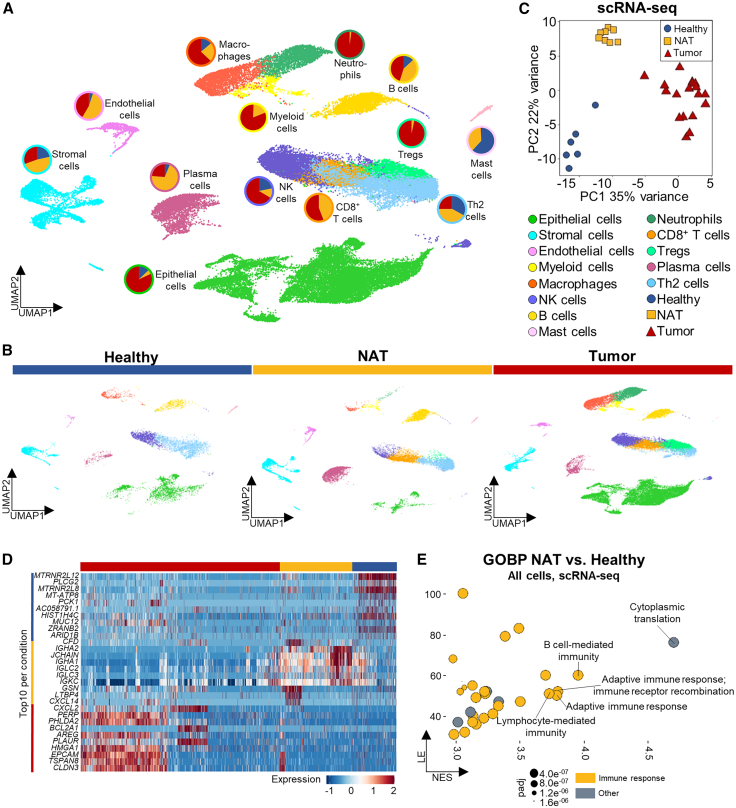
Healthy and normal adjacent colon tissue differs in cell type distribution and gene expression profile ScRNA-seq data of healthy colon material (GSE214695^9^) was integrated and harmonized with a dataset containing NAT and colorectal tumors (GSE132465^10^). The final dataset comprised 8,190 healthy, 13,628 NAT, and 37,289 tumor cells with 6 samples from healthy individuals, 18 primary CRC samples and 8 matched normal mucosa samples. (A) UMAPs illustrating thirteen distinct cell types identified in the dataset. Pie charts indicate the proportion of cells originating from healthy (blue), NAT (orange), and tumor (red) tissue per cell type. (B) UMAPs segregated into healthy, NAT, and tumor samples, demonstrating differences in cell type composition. (C) PCA plot of pseudo-bulk scRNA-seq data depicting the similarities between samples based on expression patterns in healthy (●; *n* = 6), NAT (▪; *n* = 8) and tumor (▲; *n* = 18). (D) Heatmap illustrating the top ten DEGs in healthy, NAT, and tumor cells (log_2_FC > 0.7, padj <0.05; blue: low expression, red: high expression). (E) Gene ontology of biological processes (GOBP) in NAT compared to healthy tissue from all cells reveals that the key differences are linked to immune response-associated processes (yellow). NES: normalized enrichment score, LE: leading edge, dot size inversely proportional to the adjusted *p* value.

To elucidate unique and shared features between healthy tissue and NAT, scRNA-seq datasets containing healthy colon material, NAT, and colorectal tumors were integrated and harmonized.^9^^,^^10^ In addition, we also integrated and harmonized independent scRNA-seq datasets for validation purposes, enabling us to confirm the robustness of our findings across different cohorts. We quantitatively assessed the effectiveness of dataset integration and batch correction using silhouette analysis, suggesting robust clustering quality and indicating that biological differences were preserved while batch effects were effectively reduced (Figure S6). Based on marker gene expression, cells were classified into various populations, including epithelial, stromal, endothelial, myeloid, and diverse immune regulatory cells (Figures 1A, 1B, S1A, S1B, S4A, and S4B; Table S1).

In alignment with bulk RNA-seq findings, dimensionality reduction through principal component analysis (PCA) clearly differentiated healthy samples from NAT and tumors (Figures 1C, S1C, and S4C). As anticipated, deconvolution of bulk RNA-seq data provided limited insights into the cell type composition (Figure S1D). Visualizing the cell populations per condition revealed a substantial diversity of immune regulatory cells within NAT and tumor cohorts. Notably, the healthy population exhibited only sporadic presence of neutrophils, Tregs, myeloid, and CD8-positive T cells.

Upon identifying differentially expressed genes (DEGs; Figures 1D and S4D), gene ontology analysis was performed. Similar to tumor samples, NAT displayed an upregulation of immune response-associated genes compared to healthy material (Figures 1E and S4E, Tables S2 and S3). Collectively, these observations reinforce previous findings that healthy and normal adjacent tissues are markedly distinct at the gene expression level and highlight their substantial differences in cell type composition.

### Transcriptional differences between healthy and normal adjacent cells are apparent across all cell types

**Figure 2:**
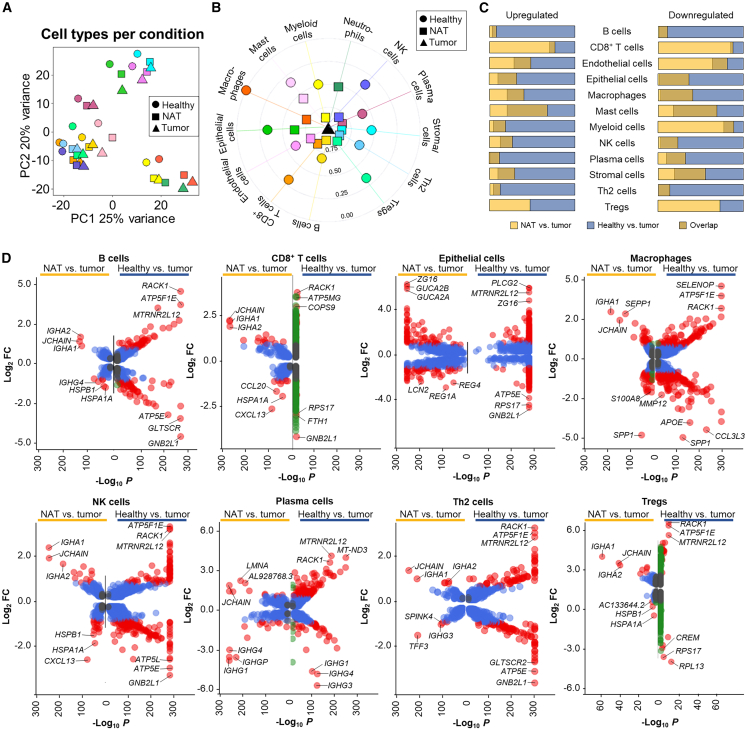
Major differences between healthy tissue, NAT and tumor emerge when deciphering cell type-specific transcriptional differences (A) PCA plot of pseudo-bulk scRNA-seq data illustrating the distinct expression patterns in healthy, NAT, and tumor cells for all cell types. NAT frequently appears in a state intermediate between healthy and tumor. (B) Based on PCA, distances from NAT and healthy cells to tumor cells were determined for each cell type. Values were normalized to the tumor condition and visualized in a circular plot. NAT consistently appears in an intermediate position between healthy and the central tumorous state. Neutrophils were not detected in healthy samples. (C) DEGs were identified by comparing NAT and healthy cells to tumor cells, respectively (“NAT vs. tumor” and “healthy vs. tumor”). These DEGs were categorized into upregulated and downregulated genes, subsequently overlapped and visualized in a Venn diagram-like graph. Log_2_FC > 0.7 or <0.7, respectively, padj <0.05. (D) Double volcano plots, showing DEGs in NAT vs. tumor and healthy vs. tumor per cell type (gray: not significant; green: log_2_FC > 1 or <1, respectively; blue: padj <0.05; red: log_2_FC > 1 or <1, respectively, padj <0.05). In B cells, epithelial cells, macrophages, NK, plasma, and Th2 cells, the transcriptional differences between healthy and tumor are substantially larger compared to the differences between NAT and tumor.

When decomposing all cell populations into healthy, NAT, and tumor, it became evident that healthy cells are clearly unique from normal adjacent and tumor cells at the gene expression level (Figures 2A and S4D). Interestingly, when normalizing the expression profile of each cell type to the tumor profile, NAT cells often exhibited gene expression patterns that are intermediate between those of tumor cells and healthy cells (Figure 2B). We observed that macrophages, Tregs, and CD8-positive T cells exhibited the most pronounced differences between healthy and NAT. This could be also observed in the validation dataset (Figure S4G). Next, we determined differential gene expression and enriched gene sets for all cell types when comparing healthy and NAT cells to tumor cells, respectively (Figures 2C and 2D, Tables S4, S5, S6, S7, S8, S9, S10, S11, S12, S13, S14, S15, S16, S17, S18, S19, S20, S21, S22, S23, S24, S25, S26, S27, S28, S29, S30). The DEGs from healthy vs. tumor and NAT vs. tumor were compared to each other and revealed relatively low overlap suggesting a low similarity of the healthy and NAT state. In addition, in the majority of cell types, the transcriptomic differences between healthy and tumor were generally larger than those between NAT and tumor. Collectively, our results suggest that NAT exhibits gene expression patterns with greater similarity to tumor cells compared to healthy tissue across multiple cell types. Among the analyzed cell types, macrophages exhibited the most pronounced transcriptomic differences between healthy control tissue and NAT (Figure 2B). Gene set enrichment analysis (GSEA) revealed significant enrichment of biological processes such as humoral immune response, phagocytosis recognition, and cell recognition in macrophages from NAT samples. These findings suggest a more immunologically active transcriptional profile in macrophages within NAT compared to those from healthy controls (Table S22). Moreover, these findings suggest that NAT may exhibit a distinct gene expression profile that reflects a complex interplay of cellular and molecular changes, some of which may be associated with tumorigenic processes.

### NAT-derived epithelial cells display a hybrid phenotype

**Figure 3:**
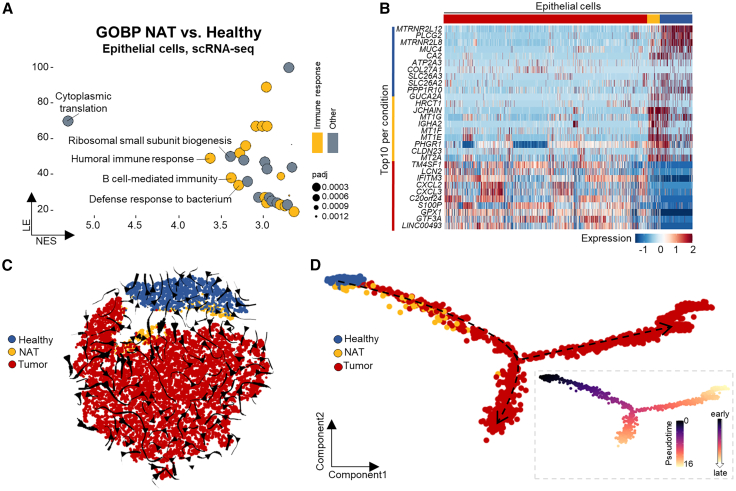
NAT-derived epithelial cells display an “intermediate” profile between healthy and tumor (A) Gene ontology of biological processes (GOBP) in NAT versus healthy tissue, focusing exclusively on epithelial cells, reveals that the key differences are linked to immune response (yellow). NES: normalized enrichment score, LE: leading edge, dot size inversely proportional to the adjusted *p* value. (B) Heatmap depicting the top 10 DEGs in healthy, NAT, and tumor epithelial cells (log_2_FC > 0.7, padj <0.05). (C) Trajectory inference of healthy, NAT and tumor cells was determined via RNA velocity using scVelo and algorithms published by La Manno et al.^11^ (D) The developmental dynamics of epithelial cells were further evaluated using monocle2,^12^ indicating early (purple) and late (yellow) fates (right image), with the developmental direction marked by an arrow.

As colorectal tumorigenesis results from a progressive transformation of epithelial cells, we focused our subsequent analyses on the epithelial population within the scRNA-seq data. 31.1%, 7.8%, and 43.2% of cells were found to be of epithelial origin in healthy tissue, NAT, and tumor, respectively. Interestingly, epithelial cells in NAT exhibited distinct gene expression profiles compared to healthy tissue, including the upregulation of genes involved in immune response (Figures 3A, 3B, S5A, and S5B; Tables S21 and S31). The identified up- and downregulated genes also showed an overlap with those in the validation dataset (Figure S5C and Table S34).

Our previous findings suggested that NAT cells occupy an intermediate state between normal and cancer. However, further analysis revealed that NAT exhibits cellular diversity, encompassing both healthy and tumor-associated cell populations. This cellular diversity explains the observation that many genes are expressed at levels between those observed in healthy and tumor cells. Indeed, trajectory inference analysis revealed a more nuanced picture. While healthy cells were predicted to represent the origin, both RNA velocity (Figure 3C) and pseudotemporal development via monocle (Figure 3D) indicated that NAT cells exhibit a heterogeneous trajectory. Notably, a subpopulation of NAT cells clustered with healthy cells, while another subpopulation clustered with early tumor cells along the pseudotime trajectory, suggesting that NAT harbors cells at various stages of transformation.

### NAT exhibits tumor-like copy number variations

**Figure 4:**
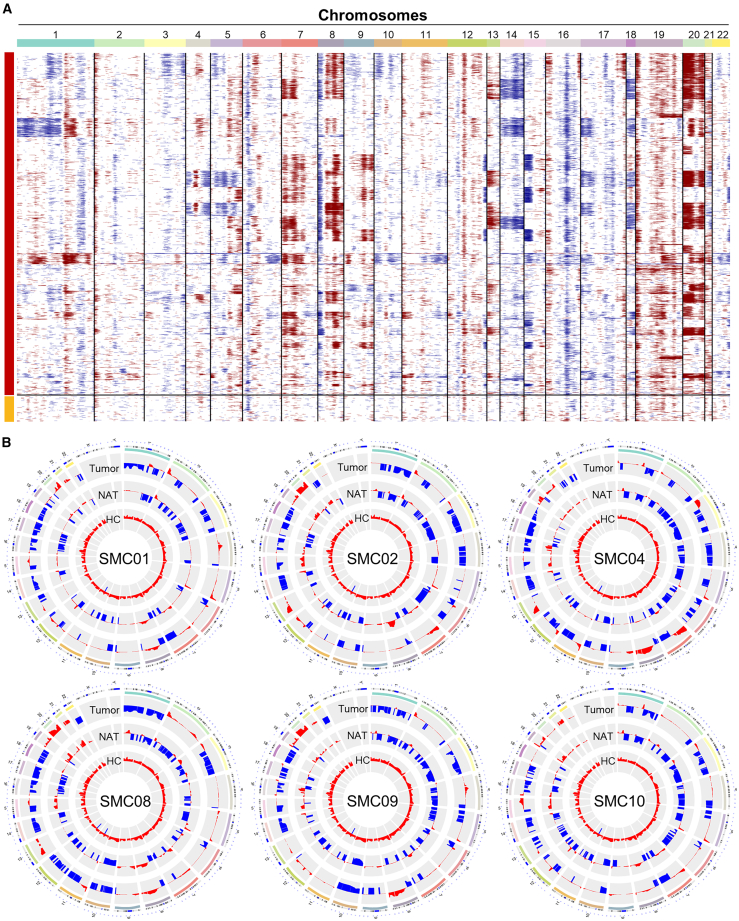
NAT-derived epithelial cells harbor tumor-like CNVs Genome-wide determination of copy number variations using the inferCNV package referencing healthy epithelial cells. (A) Average signal across all CRC patients revealed common CNVs in NAT (yellow) and tumor cells (red). (B) Patient-wise analysis of CNVs in NAT and tumors.

Next, to determine whether similarities and differences between tissue types were only present at the transcriptomic level, we analyzed copy number variations (CNVs) using healthy cells as a reference (Figures S2A and S7). Interestingly, CNV analysis of the average signal across all patients revealed that NAT cells not only resemble tumor cells at the gene expression level but also exhibit genetic alterations characteristic of colorectal cancer, potentially indicating tumor-associated properties (Figures 4A and S5E). The resemblance between NAT and tumor cells becomes even more striking when CNVs are examined at the individual patient level, where distinct tumor-associated alterations emerge (Figure 4B). This observation, combined with the unique gene expression profile and the presence of tumor-associated CNVs in NAT, suggests that NAT comprises a heterogeneous population of cells, including both healthy and tumor-associated subsets, contributing to its apparent “intermediate” state.

### Identification of marker genes to differentiate normal epithelial and tumor cells

**Figure 5:**
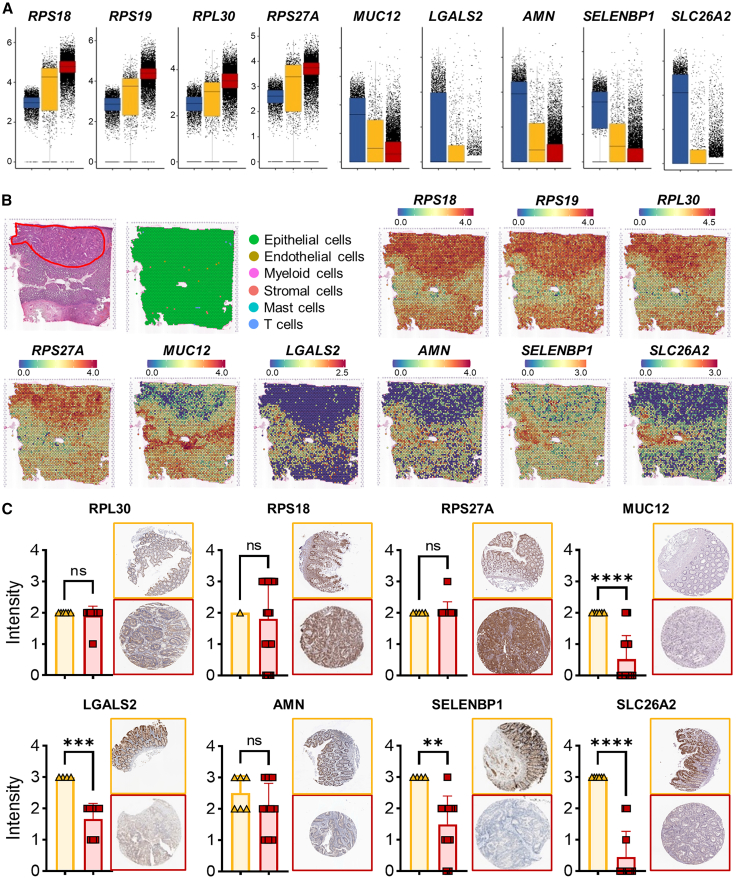
Identification of genes to distinguish between healthy and tumor tissues (A) Normalized expression of candidate genes within healthy (blue), normal adjacent (yellow), and tumor (red) tissues as determined using scRNA-seq. ∗p˂0.05, ∗∗p˂0.01, ∗∗∗p˂0.001, ∗∗∗∗p˂0.0001. One-way ANOVA. (B) Representative spatial transcriptomics results visualizing the detected cell types, hematoxylin and eosin (H&E) staining with marked tumor regions (red), and normalized expression levels of nine markers (blue: low expression, red: high expression^13^). (C) Staining intensity and representative immunohistochemical stainings for the respective markers on CRC (red) and normal control tissues (orange; 0: negative, 4: high staining intensity; Student’s *t* test). Data were retrieved from the Human Protein Atlas.^14^

Our findings suggest that NAT is not an appropriate control tissue when analyzing tumor samples due to a distinct gene expression profile across all cell populations compared to healthy controls, as well as the presence of CRC-associated CNVs and gene expression patterns in epithelial cells. These findings highlight the need for accurate identification of molecular changes within tumor cells which are also frequently present in NAT to distinguish them from healthy cells. Thus, differential gene expression analysis was conducted using both, scRNA-seq and spatial transcriptomics data. The ribosomal protein-encoding genes *RPS19*, *RPL30*, *RPS18*, and *RPS27A* displayed elevated expression in colorectal tumors compared to healthy tissue, while *MUC12*, *LGALS2*, *AMN*, *SELENBP1*, and *SLC26A2* were expressed at low levels (Figure 5A). Importantly, the expression of these genes in NAT showed intermediate levels, reflecting the heterogeneity of this tissue. Notably, high expression of genes upregulated in tumors (and NAT) and low expression of genes downregulated in tumors (and NAT) were associated with poor prognosis in CRC patients (Figure S3).

To evaluate the ability of the identified markers to highlight cells exhibiting tumor-like characteristics, we analyzed their expression patterns in spatial transcriptomics data encompassing both tumor tissue and NAT.^13^ Despite the small tissue size analyzed in this approach, we were able to confirm the distinct enrichment of the genes identified in scRNA-seq (Figures 5B and S2B). Notably, within NAT regions, we observed subpopulations of cells exhibiting gene expression patterns with features associated with tumorigenesis, further supporting the presence of cellular heterogeneity within NAT. Moreover, a majority of genes that were differentially expressed between tumor and NAT in the scRNA-seq dataset showed the same direction of regulation in the spatial transcriptomic data, supporting the overall consistency across platforms (Figure S2C and Table S33). As a complementary approach, immunohistochemical staining on colorectal tumors and normal controls were evaluated, which indeed displayed similar results (Figure 5C), namely, significant differences in markers that could differentiate between healthy and tumor tissue. In particular, staining of MUC12, LGALS2, SELENBP1, and SLC26A2 enabled a clear identification of tissue identity. In conclusion, we have identified a gene panel that can effectively distinguish between healthy tissue and tissue with gene expression profiles associated with tumorigenesis, including some regions within NAT. This gene panel may have potential applications for future research and diagnostic purposes.

### Gene expression patterns in pseudotime and tissue organization

**Figure 6:**
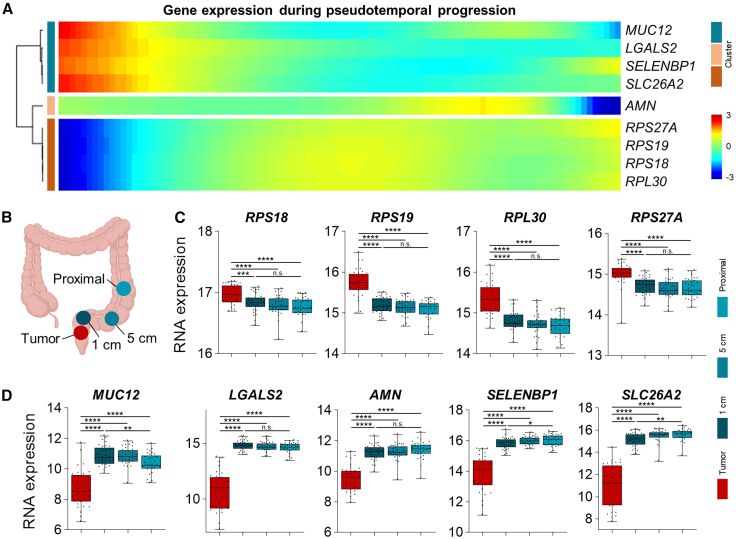
Expression of marker genes regarding to pseudotemporal allocation and tissue organization (A) Heatmap depicting genes differentially expressed during pseudotemporal progression (q-val <0.1, blue: low expression, red: high expression). (B) Schematic representation of the biopsy locations in a publicly available microarray dataset (GSE90627^15^). (C) Expression of four selected late-increasing and (D) five late-decreasing markers within tumor (red) and at biopsy distances of 1 cm (dark blue), 5 cm (blue), and the proximal end (light blue). ∗p˂0.05, ∗∗p˂0.01, ∗∗∗p˂0.001, ∗∗∗∗p˂0.0001. One-way ANOVA.

To further investigate gene expression dynamics, we analyzed the expression patterns of the identified marker genes along the pseudotemporal trajectory of the epithelial population. While the presence of both healthy-like and tumor-like cells within NAT presents challenges for traditional pseudotime analysis that assumes a linear progression from healthy to tumor tissue, we employed a modified approach to evaluate the expression of the markers we identified within the epithelial population. This analysis revealed that genes encoding ribosomal proteins (*RPS19*, *RPL30*, *RPS18*, and *RPS27A*) showed low expression in clusters enriched for cells with gene expression profiles similar to healthy tissue (early fate) and increased expression in clusters enriched for cells with gene expression profiles associated with tumorigenesis (late fate). Conversely, genes such as *MUC12*, *LGALS2*, *AMN*, *SELENBP1*, and *SLC26A2* exhibited decreased expression in clusters enriched for cells with gene expression profiles associated with tumorigenesis compared to clusters enriched for cells with gene expression profiles similar to healthy tissue (Figure 6A). These findings demonstrate the presence of distinct gene expression programs within the epithelial compartment, reflecting the heterogeneity observed within NAT.

To assess the clinical relevance of our findings, we analyzed publicly available gene expression values^15^ from left-sided colorectal tumors as well as biopsies taken at 1 cm and 5 cm distances from the tumor lesion, in addition to biopsies collected from the proximal end of the colon (Figure 6B). We hypothesized that the identified gene expression signatures could reflect the spatial proximity to the tumor. Indeed, our analysis revealed a gradient of gene expression along the colorectal axis, with the expression of ribosomal protein-encoding genes gradually decreasing and the expression of other genes, except for *LGALS2*, gradually increasing with increasing distance from the tumor (Figure 6C). These findings suggest that the identified gene expression signatures exhibit spatial gradients along the colorectal axis, reflecting the proximity to the tumor. This observation may have implications for understanding the spatial organization of the tumor microenvironment as well as for surgical planning and risk assessment in colorectal cancer.

### Targeting tumor-like cells within NAT to reduce CRC recurrence

**Figure 7:**
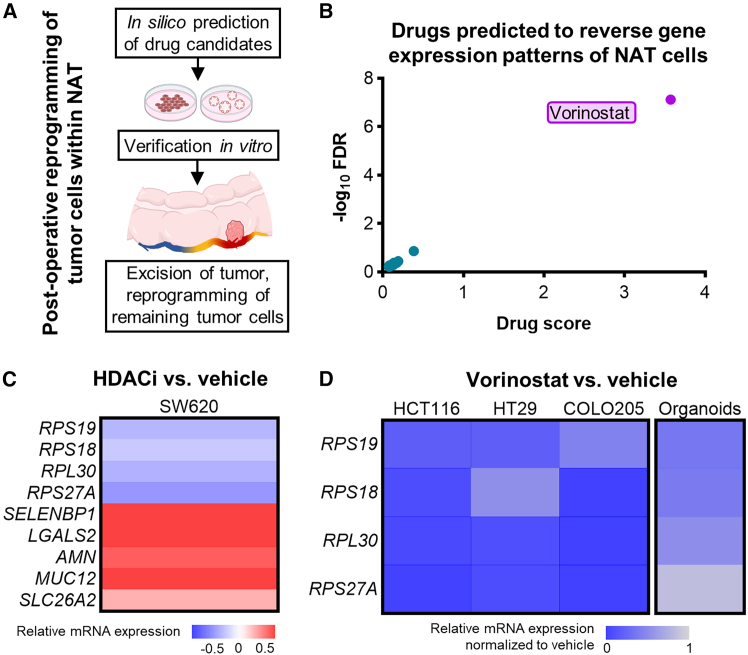
Pharmaceutical reprogramming of tumor cells (A) Schematic representation of the pharmacological reprogramming of tumor cells *in vitro* and remaining tumor cells within NAT after surgery. (B) Using the ASGARD package,^18^ the most promising drugs to reprogram expression patterns of NAT epithelial cells were identified. Vorinostat displayed the highest drug score. (C) RNA-seq data from the human CRC cell line SW620 were analyzed following treatment with the HDACi CXD101 (GSE158164^16^). The relative mRNA expression of marker genes compared to controls is displayed in a heatmap (red: upregulated, blue: downregulated). (D) Human CRC cell lines (*n* = 3) and human colon organoids (*n* = 5) were treated with vorinostat in three independent experiments. Gene expression levels were compared to vehicle-treated controls using RT-qPCR.

To improve patient outcomes in CRC, innovative strategies are needed to address the persistent issue of local recurrence. Our findings demonstrate that NAT exhibits features reminiscent of tumors, suggesting that reprogramming tumor cells within NAT after surgery could be a promising complementary strategy to reduce recurrence and improve overall survival (Figure 7A). To identify potential therapeutic agents for this approach, we employed the ASGARD analysis pipeline which leverages single-cell data to repurpose drugs. Based on the transcriptomic differences between epithelial cells in normal adjacent and healthy tissues, this tool identified the histone deacetylase (HDAC) inhibitor (HDACi) vorinostat as a potential therapeutic candidate for modulating gene expression of remaining tumor cells in NAT (Figure 7B and Table S32). As a proof of concept, we analyzed the transcriptomic changes induced by HDACi treatment in the human CRC cell line SW620 using bulk RNA-seq data.^16^ Indeed, HDAC inhibition resulted in a downregulation of genes encoding ribosomal proteins and an upregulation of the inverse signature (Figure 7C). Previous research has demonstrated the feasibility of targeting ribosomal proteins, which are essential for increased biosynthesis needs of tumors, in cancer therapy.^17^ Therefore, we aimed to directly verify the ability of the proposed HDACi vorinostat to reduce the expression of the four ribosomal genes *in vitro*, indicating a shift toward a healthier tissue state. Indeed, vorinostat effectively reduced the expression of *RPS19*, *RPL30*, *RPS18*, and *RPS27A* in HCT116, HT-29, and COLO205 cells compared to vehicle-treated controls (Figure 7D). Furthermore, we sought to validate these results using organoids derived from primary colorectal tumors. Our experiments demonstrated that vorinostat treatment similarly reduced the expression of ribosomal genes in these organoid models, reinforcing the potential of this drug to reprogram tumor cells, including those present in NAT, toward a more “healthy” state, thereby reducing the risk of local recurrence.

## Discussion

This study underscores the critical distinction between NAT and healthy tissue in the context of colorectal cancer. While NAT is often considered a suitable control in cancer research due to its histological normalcy, our findings reveal significant molecular deviations across cell types that challenge this assumption and strengthen previous bulk RNA-seq-based studies.^3^^,^^5^^,^^6^^,^^19^ Notably, we identified marker genes for distinguishing between healthy and tumor cells during pathological evaluations.

Our identification of DEGs between healthy, normal adjacent and tumor cells provides a valuable resource for future CRC research, particularly for studies that utilize NAT as a control without incorporating healthy tissue. Our and previous studies^3^^,^^5^^,^^6^^,^^19^ raise concerns that using NAT as a control may lead to misleading interpretations as the unique characteristics of tumors could be overlooked by the shared features with NAT. While completely healthy tissue would serve as a more appropriate control, its acquisition presents practical challenges due to the routine removal of NAT during tumor resection. In addition, even though the quality of life in healthy volunteers is not significantly affected by endoscopic procedures, endoscopy and the collection of mucosa are not completely risk-free.^20^ Importantly, our and similar studies do not imply that all studies employing NAT as a control are fundamentally flawed. They rather highlight the need for careful consideration of the control tissue used in research. As the landscape of cancer research evolves, our findings encourage a re-evaluation of methodologies to ensure the validity and reliability of study results.

In fact, similar discoveries in the past have already highlighted the importance of clinical routines regarding the extent of NAT excision during tumor resections. For instance, it was demonstrated that carcinoembryonic antigen (CEA) expression extends 5 cm from the peritumoral area in CRC.^21^ In a study that identified profound transcriptomic differences between healthy and NAT using bulk RNA-seq, NAT samples from a minimum of 10 cm away from the tumor resection margin were collected.^5^ These findings underscore the importance of careful surgical margin assessment to minimize the risk of local recurrence, as the presence of cellular and molecular alterations within NAT may contribute to tumor progression.

In agreement with previous studies which described an enrichment of wound response-associated pathways^22^^,^^23^ in NAT, our GO analyses revealed an increase in immune regulatory processes when comparing NAT to healthy tissue. The possibility that cancerous cells within NAT may have tumor-initiating capabilities is an important area for future research, as it may reveal targets for therapeutic intervention and strategies to enhance patient prognosis. The exploration of NAT has sparked discussion since Slaughter et al. first introduced the concept of “field cancerization”.^24^ This theory posits that carcinogenesis occurs gradually, with the accumulation of genetic and epigenetic alterations over time. As a result, NAT may exhibit cellular and molecular heterogeneity, including cells with features associated with tumorigenic processes.

Intriguingly, the patient-specific analysis of CNVs within NAT revealed consistent tumor-like genetic alterations, reinforcing the notion of its heterogeneous cellular composition and distinct molecular landscape compared to healthy tissue. Future investigations are warranted to fully elucidate the clonal dynamics and functional consequences of these CNV patterns within NAT. While our CNV predictions are based on transcriptional inference from scRNA-seq data, the observed alterations in NAT are consistent with previous reports of molecular abnormalities in tumor-adjacent tissue. Nonetheless, orthogonal validation using DNA-based CNV profiling or multimodal single-cell approaches would be valuable to further strengthen these findings.

In order to reprogram cancerous cells within NAT tissue, we unveiled the HDAC inhibitor vorinostat by ASGARD analysis. Vorinostat effectively downregulated the expression of ribosomal proteins in both cell lines and organoid models, suggesting its potential to reprogram tumor and NAT toward a more “healthy” state. This approach holds promise for reducing the risk of local recurrence in colorectal cancer. In fact, previous research has demonstrated the feasibility of targeting ribosomal proteins in cancer therapy.^17^ Elucidating vorinostat’s effects on primary NAT-derived organoids should be a key focus of future investigations. Complementary to this, elucidating the intricate cell-cell interactions within tumor, normal adjacent, and healthy tissues will provide crucial mechanistic insights into tumor progression and potential therapeutic avenues. Apart from our own study, researchers observed a beneficial effect of vorinostat in metastatic CRC.^25^ However, previous phase I and II studies have reported significant adverse effects associated with vorinostat. Given the low tolerability observed in patients with colorectal, breast, thyroid, cutaneous T cell lymphoma, diffuse large B cell lymphoma, or non-small-cell lung cancer,^26^ future research should aim to investigate the efficacy of more specific and potentially better-tolerated HDAC inhibitors. We have generated preliminary data testing several structurally distinct HDAC inhibitors which indicate differential effects on transcriptomic profiles. We will further investigate the specific HDAC isoforms involved and evaluate compounds with improved specificity and tolerability in future studies.

For our bioinformatics analyses, two scRNA-seq datasets were integrated and harmonized after merging to reduce batch effects. While superior performance of Harmony to previously published algorithms was demonstrated,^27^ disparities between the two datasets, such as variations in donor age, sampling site, processing protocols, or sequencing platforms, cannot be completely excluded. While cross-cohort integration carries inherent challenges, our silhouette metric supports the conclusion that the applied batch correction strategy removed batch effects and did not introduce substantial technical distortion. To confirm our findings, we validated our analysis in a second scRNAseq dataset. Furthermore, the differential expression of the gene panel we identified was observed in multiple bulk datasets. Future studies are needed to address the difference between healthy and NAT by sequencing all three tissue types in the same experimental setting.

Together, our findings highlight the distinct transcriptomic state and CNV profile of NAT compared to both healthy and tumor tissues. We identified a gene panel that accurately differentiates these tissue types, offering valuable insights for researchers studying CRC. By delineating the specific gene expression profiles at a single-cell level that distinguish NAT from tumor and healthy tissues, our study challenges the traditional use of NAT as a standard control. Understanding the molecular landscape surrounding tumors is crucial for interpreting research findings accurately. Our data serve as a reference point for researchers investigating tumor biology, emphasizing the importance of accounting for the complexities introduced by the use of NAT in experimental designs.

### Limitations of the study

This study integrated two independent scRNA-seq datasets using Harmony to attenuate batch effects. Nonetheless, residual inter-cohort variability, arising from differences in donor demographics, sampling procedures, tissue processing, or sequencing platforms, cannot be entirely excluded and may influence downstream analyses.

The information about sex was not included for *in vitro* experiments due to the small number of samples.

## Resource availability

### Lead contact

Requests for further information and resources should be directed to and will be fulfilled by the lead contact, PD Dr. Robyn Laura Kosinsky (robynlaura.kosinsky@bosch-health-campus.com).

### Materials availability

This study did not generate new unique reagents.

### Data and code availability

#### Data

This paper analyzes existing, publicly available data, accessible at Gene Expression Omnibus (GEO) database (https://www.ncbi.nlm.nih.gov/geo/): GSE95132, GSE158164, GSE90627, GSE214695, GSE214695, GSE132465, GSE178341, GSE231993.

#### Code

All custom scripts and code used for the analysis in this study will be made publicly available at the following GitHub repository: https://github.com/bhc-rbct. The repository will include documentation to facilitate reproducibility and reuse of the methods presented.

#### Additional information

Any additional information required to reanalyze the data reported in this paper is available from the lead contact upon request.

## Acknowledgments

This work was supported by the 10.13039/501100001646Robert Bosch Stiftung and the Monika Kutzner Stiftung Berlin.

## Author contributions

R.L.K. and P.R. designed the study with input from D.S. Bioinformatics analyses were performed by P.R., D.S., and S.M.S. with the help of O.M.B., C.P., and R.L.K. Cell line experiments were carried out by P.R. and organoid cultures by J.K., D.M., and T.M. Clinical samples were provided by M.H.D. Survival curves were generated by J.A.F. The manuscript was written by R.L.K. and P.R. with support of D.S., O.M.B., and X.W. All authors have read and approved the final version of the article, including the authorship list.

## Declaration of interests

The authors declare no conflict of interest.

## Declaration of generative AI and AI-assisted technologies in the writing process

During the preparation of this work the author(s) used ChatGPT (OpenAI) for language editing and stylistic corrections. After using this tool, the author(s) reviewed and edited the content as needed and take(s) full responsibility for the content of the publication.

## STAR★Methods

### Key resources table

**Table:** 

REAGENT or RESOURCE	SOURCE	IDENTIFIER
**Biological samples**		

Patient-derived CRC organoids	Dr. Margarete Fischer-Bosch Institute of Clinical Pharmacology, Bosch Health Campus	N/A

**Deposited data**		

Bulk RNAseq data	Gene Expression Omnibus (GEO) database	GSE95132, GSE158164
Microarray data	Gene Expression Omnibus (GEO) database	GSE90627
scRNAseq data	Gene Expression Omnibus (GEO) database	GSE214695, GSE132465, GSE178341, GSE231993

**Experimental models: Cell lines**		

Human CRC cell line HCT116	ATCC	CCL-247
Human CRC cell line HT29	ATCC	HTB-38
Human CRC cell line Colo205	ATCC	CCL-222

**Oligonucleotides**		

Primer: RNA18SN1 forward 5′- ACTGCCATTAAGGGTGTGGG-3′	This paper	N/A
Primer: RNA18SN1 reverse 5′- CAGGTCTTCACGGAGCTTGT-3′	This paper	N/A
Primer: RPS19 forward 5′-GCCTGGAGTTACTGTAAAAGACG-3′	This paper	N/A
Primer: RPS19 reverse 5′-CCCATAGATCTTGGTCATGGAGC-3′	This paper	N/A
Primer: RPL30 forward 5′-TACGTCCTGGGGTACAAGCA-3′	This paper	N/A
Primer: RPL30 reverse 5′-AAAGCTGGGCAGTTGTTAGC-3’	This paper	N/A
Primer: RPS18 forward 5′-TGTGGTGTTGAGGAAAGCA-3′	This paper	N/A
Primer: RPS18 reverse 5′-CTTCAGTCGCTCCAGGTCTT-3′	This paper	N/A
Primer: RPS27A forward 5′-CTGGAAGATGGACGTACTTTGTC-3′	This paper	N/A
Primer: RPS27A reverse 5′-CGACGAAGGCGACTAATTTTGC-3′	This paper	N/A

**Software and algorithms**		

edgeR (v3.38.4).	Yunshun Chen	https://bioconductor.org/packages/release/bioc/html/edgeR.html
DESeq2 (v1.36.0 and v2.11.40.8)	Michael Love	https://bioconductor.org/packages/release/bioc/html/DESeq2.html
GSEABase (v1.58.0)	Martin Morgan	https://bioconductor.org/packages/release/bioc/html/GSEABase.html
ggplot2 (v3.4.0 and v3.4.1 and v3.4.3)	Wickham H	https://ggplot2.tidyverse.org/
MuSiC (v1.0.0)	Wang et al.^28^; Fan et al.^29^	https://github.com/xuranw/MuSiC
FastQC (v0.74)	Andrews S	https://github.com/s-andrews/FastQC
FASTQ Trimmer (v1.1.5)	Gordon A	https://github.com/auerlab/fastq-trim
fastp (v0.23.2)	Shifu Chen	https://github.com/OpenGene/fastp
RNA STAR (v2.7.10b)	Alexander Dobin	https://github.com/alexdobin/STAR?tab=readme-ov-file
htseq-count (v0.9.1)	Anders S	https://htseq.readthedocs.io/en/release_0.10.0/overview.html
Galaxy Project Europe platform	Afgan E	https://github.com/galaxyproject
RStudio (v4.2.1)	R project	https://github.com/rstudio/rstudio
RColorBrewer (v1.1-3)	Erich Neuwirth	https://cran.r-project.org/web/packages/RColorBrewer/index.html
PCAtools (v2.8.0).	Kevin Blighe	https://github.com/kevinblighe/PCAtools
Seurat (v5.0.0 and v4.3.0.1)	Butler et al.^30^; Stuart et al.^31^	https://github.com/satijalab/seurat
SeuratObject (v4.1.3)	Butler et al.^30^; Stuart et al.^31^	https://github.com/satijalab/seurat-object
harmony (v0.1.1)	Korsunsky et al.^27^	https://github.com/immunogenomics/harmony
monocle2 (v2.18.0)	Qiu et al.^12^	https://bioconductor.org/packages/release/bioc/html/monocle.html
PhyloVelo	Wang et al.^32^	https://github.com/kunwang34/PhyloVelo
Python (v3.8.15)	Python Software Foundation	https://www.python.org
matplotlib (v. 3.9.2)	J.D. Hunter	https://matplotlib.org/
infercnv (v1.14.2)	Timothy et al.^33^	https://github.com/broadinstitute/infercnv/wiki
OmicCircos (v1.36.0)	Ying Hu	https://bioconductor.org/packages/release/bioc/html/OmicCircos.html
msigdbr (v7.5.1)	Dharmesh D	https://www.bioconductor.org/packages/release/data/experiment/html/msigdb.html
clusterProfiler (v4.4.4)	Guangchuang Yu	https://bioconductor.org/packages/release/bioc/html/clusterProfiler.html
GraphPad Prism (v9.4.1)	GraphPad Software	https://www.graphpad.com/
ASGARD package (v1.0.0)	He et al.^18^	https://github.com/lanagarmire/Asgard
limma package (v3.52.4).	Rirchie et al. (2015)	https://bioconductor.org/packages/release/bioc/html/limma.html
bioVenn	Hulsen et al. (2008)	https://www.biovenn.nl/index.php

**Other**		

Code availability	This paper	https://github.com/bhc-rbct

### Experimental model and study participant details

#### Cell lines and primary cultures

Human HCT116 and HT-29 colorectal cancer cells were grown in McCoy’s medium and COLO 205 cells in RPMI-1640 medium supplemented with 10% fetal bovine serum, 100 units/ml of penicillin, and 100 μg/mL of streptomycin at 37°C in a 5% CO_2_ atmosphere. All cell lines were authenticated using STR verification and tested negative for mycoplasma contamination regularly. Primary colorectal cancer samples were obtained from patients who underwent surgery at the Robert-Bosch-Hospital, Stuttgart. The study was approved by the Ethical Committee at the University Tübingen (696/2016BO2) and written informed consent was obtained. Residual tissue samples, which were not used for pathological routine examination, were transferred to the laboratory for cell isolation within a maximum of 8 h after surgery. Organoid culture was performed as previously described.^34^ Cells were treated with 3 μM vorinostat (Adooq) for 24 h. As a negative control, an equal volume of DMSO was added to the cells.

### Method details

#### Data acquisition

This study utilized six publicly available datasets including bulk RNA-seq, scRNA-seq, and spatial transcriptomics data to investigate gene expression profiles across healthy, normal adjacent tissue (NAT), and colorectal cancer (CRC) samples. All datasets were downloaded from the Gene Expression Omnibus (GEO) database (https://www.ncbi.nlm.nih.gov/geo/) unless otherwise specified. The first bulk RNA-seq dataset, GSE95132, included 5 normal crypt, 10 NAT, and 10 colorectal cancer tumor samples. The second bulk RNA-seq dataset, GSE158164, contained gene expression profiles for CXD101-treated (*n* = 2) and untreated (*n* = 3) SW620 cells. The microarray dataset, GSE90627, represented a serial biopsy dataset from 32 rectal cancer patients. Biopsy samples were collected from four locations: directly from the tumor (0 cm), 1 cm away from the tumor, 5 cm away from the tumor, and from the proximal end of the colon.

The scRNA-seq dataset was created by combining two datasets available at GEO: GSE214695 and GSE132465. GSE214695 contained 6 samples from healthy individuals. GSE132465 included 18 primary CRC samples and 8 normal mucosa samples. The second scRNA-seq validation dataset was created by the integration of the datasets GSE178341, including 62 tumor and 26 normal adjacent samples, and GSE231993 containing 4 healthy controls. The spatial transcriptomics dataset comprised 4 CRC samples. It was obtained from the website http://www.cancerdiversity.asia/scCRLM/.

#### Processing of CRC tissue-based bulk data

The counts-per-million (CPM) of each gene within the bulk RNA-seq dataset were calculated using the cpm function in edgeR (v3.38.4). Lowly expressed genes with CPM values less than 0.5 and expressed in only one sample were removed from the dataset. The counts were converted to a DGEList object, their log_2_ values calculated, normalized, and the multidimensional scaling plot of the distances between gene expression profiles was visualized using edgeR. Differential gene expression analyses between the healthy, NAT, and tumor groups were performed with DESeq2 (v1.36.0). Subsequently, GSEABase (v1.58.0) was used on the output of the differential gene expression analyses to identify enriched gene sets in each group. For the analysis of tumor and NAT markers at different distances from the tumor, the microarray RNA-seq dataset (GSE90627) was utilized. The ggplot2 package (v3.4.0) was employed to illustrate the log2-normalized gene expression levels of selected potential markers. The GSE95132 bulk RNA-seq dataset was deconvoluted using the workflow established by MuSiC (v1.0.0).^28^^,^^29^ The scRNA-seq dataset was used in training the deconvolution algorithm, along with selected canonical cell markers, to estimate cell types in all samples of the bulk RNA-seq dataset. Estimated proportions of the selected cell types were visualized using the Jitter_Est and Boxplot_Est functions of the MuSiC package. To determine the distribution of cell types within the healthy and tumor groups, the normalized counts of each sample were summed within each group. The combined counts matrix was then used with the scRNA-seq dataset in the MuSiC package, along with the same canonical cell markers, to calculate the estimated cell distribution of the selected cell types across the groups. The graphical illustrations of the cell distributions in each sample and each group were generated using the ggplot2 package (v3.4.0). Batch correction for the variance analysis between the bulk RNA-seq and scRNA-seq datasets was measured by dividing the mean expressions of bulk RNA-seq samples by the mean expression of scRNA-seq samples and multiplying the resultant with each gene. The PCA plot was generated using the ggplot2 package.

#### Processing of CRC cell line-based bulk data

The RNA-seq data of SW602 cells (GSE158164) were analyzed using the Galaxy Project Europe platform. FastQC (v0.74+galaxy0) was used for quality control of the FASTQ files, with a Kmer length of 7. Low-quality sequences were removed by the FASTQ Trimmer (v1.1.5), followed by a second round of quality control. Some of the remaining reads were observed to have missing quality scores, and these reads were filtered using the fastp (v0.23.2+galaxy0) tool. Reads were aligned to the hg38 human reference genome using RNA STAR (v2.7.10b+galaxy4). Overall counts were summed to aligned reads using the htseq-count (v0.9.1+galaxy1) tool. Reads overlapping multiple genes were aligned using the union mode in the htseq-count tool. DEGs were determined using DESeq2 (v2.11.40.8+galaxy0). In addition to the differentially expressed genes table, the normalized counts of the genes for each sample were exported as an output in this step. The normalized counts were further analyzed in RStudio (v4.2.1) to generate heatmaps and PCA plots using ggplot2 (v3.4.1), RColorBrewer (v1.1-3), and PCAtools (v2.8.0).

#### Processing of CRC scRNA-seq data

ScRNA-seq data were downloaded from the GEO database. The Seurat package (v5.0.0)^30^^,^^31^ was used to perform integrated analyses of single cells. Genes expressed in fewer than three cells, cells that expressed fewer than 250 genes, and cells with more than 20% mitochondrial gene expression were excluded from downstream analysis in each sample. Each dataset was SCTransform-normalized, and the top 3,000 Highly Variable Genes (HVGs) across cells were selected. Datasets were integrated based on “anchors” identified between datasets before Principal Component Analysis (PCA) was performed for linear dimensional reduction. Both datasets were combined using the merge function (SeuratObject v4.1.3). The dataset was normalized and integrated based on the 3,000 most variable features. A Principal Component Analysis was performed using the RunPCA function (Seurat), and a Uniform Manifold Approximation and Projection (UMAP) was created by applying the RunUMAP function (dims = 1:40, reduction = “pca”). Finally, the dataset was harmonized by running the RunHarmony function of the harmony package (v0.1.1).

#### Silhouette analysis

To evaluate the quality of integration and clustering, we computed silhouette scores using the silhouette() function from the R package cluster, based on a pairwise distance matrix derived from the integrated expression. Cells were grouped by their cluster assignments, and distances were calculated using a precomputed distance matrix. The resulting silhouette values were visualized using the built-in plot() function, and the overall clustering quality was summarized by the average silhouette width.

#### Clustering and assignment of the cell types

Clusters were assigned by first running the FindNeighbors function of the Seurat package (v4.3.0.1) using the first 40 dimensions. Different cluster variations were calculated using the FindClusters function (Seurat v4.3.0.1) with a resolution of 1.4. For Uniform Manifold Approximation and Projection (UMAP) calculations, the RunUMAP function (dims = 1:40, reduction = “pca”) was applied. A DimPlot (Seurat v4.3.0.1) of the UMAP reduction was plotted to visualize the results. In total, 50 clusters were identified. Cell types were assigned according to the expression of previously described marker genes for epithelial cells (*EPCAM, KRT8, CDH1*), stromal cells (*COL1A1, COL1A2, COL3A1*), endothelial cells (*CLDN5, PLVAP, ID3*), Myeloid cells (*FCGR3A, HLA-DRA, S100B, CD14, CD74*), macrophages (*CD68, CD163, CLEC7A, FOLR2, PLTP, SEPP1, CCL18*), natural killer cells (NK cells; *GNLY, NKG7, IL2RB, KLRB1, KLRD1*), plasma cells (*CD79A, MS4A1, CD79B*), mast cells (*KIT, MS4A2, LTC4S, TPSB2, TPSAB1, CPA3*), neutrophils (*CSF3R, LYZ, PLAUR, FCN1, CECR1, SLC11A1*), CD8^+^ T cells (*CD8A, CD8B, GZMK*), regulatory T cells (Tregs; *FOXP3, IL2RA, CD4*), Th1 cells (*CD4, CXCR3*^*+*^*, CXCR4*^*+*^) and Th2 cells (*CD4, CXCR3*^*-*^*, CXCR4*^*+*^).

#### Principal component analysis

PCA plots were created after normalizing, scaling, and clustering the dataset. First, the two conditions “class” and “sample/cell type” were combined into a single column. Then, the expression of each gene per this variable (*n* = 29/35) was averaged. Variance stabilization was performed using the DESeq2 package (v1.36.0). PCA was plotted using the plotPCA function (DESeq2) and ggplot (ggplot2 v3.4.3). For distance calculation, the information from PC1 and PC2 was extracted using the subset function (Seurat v4.3.0.1). Subsequently, the distance between all conditions was calculated using distance matrix computation. Distance values for NAT and healthy samples were normalized based on the distance to the corresponding tumor sample. Visualization was done using the ggplot function (ggplot2 v3.4.3).

#### Pseudotime trajectory analysis

For pseudotime analysis of epithelial cells, monocle2 (v2.18.0, reduction method = DDRTree, max. components = 2, heatmaps constructed with differential gene test according to “∼sm.ns(Pseudotime)”, significance level qval<0.1, plotted with plot_pseudotime) was used.

#### Velocity

Velocity analyses have been performed using PhyloVelo^32^ in python (v3.8.15). Briefly, after manual UMAP- and cell annotation-transfer from the R-created UMAPs (see above), normalization and trajectory inference (cutoff = 0.95) were performed on the raw counts. Subsequently, velocity embedding (15 neighbors) was induced and plotted via matplotlib (v. 3.9.2), following the recommendations of Wang et al.^32^

#### Copy number variations (CNVs)

Copy number variations were calculated using infercnv (v1.14.2), following the recommendations from Tickle and colleagues.^33^ Briefly, the count matrix, annotation, and genecodes were read into R via the CreateInfercnvObject function, using the human v19 genecode provided by the Broad Institute. Subsequently, a cutoff of 0.1 (for 10X genomics datasets) was applied, and the analysis was performed without clustering, using a Hidden Markov Model (HMM)-based CNV detection algorithm. With the OmicCircos package (v1.36.0), circular plots were created on the human genome h19 to visualize duplications and deletions across the genome.

#### Differentially expressed gene analysis

DEGs between healthy, NAT, and tumor cells were calculated using the FindAllMarkers function (min.pct = 0.1, logfc.threshold = 0.25). Markers for each class were separated using the subset function (Seurat), and the top 10 genes were identified based on the log_2_FC. The top 10 genes for each class were combined, their expression was scaled, and the results were visualized using the DoHeatmap function (Seurat).

#### Gene Set Enrichment Analysis

For gene set enrichment analysis, the FindAllMarkers function (Seurat) was used to identify differentially expressed genes. A gene list was created containing the average log_2_FC values and the gene names as row names. To retrieve the ontology gene set (C5) for the analysis, msigdbr (v7.5.1) was applied (Species: *Homo sapiens*). GSEA, a function of the clusterProfiler package (v4.4.4), was performed to identify enriched gene sets. Results were filtered based on the subset GOBP (biological processes). The top 10 results were identified based on the log_2_FC (padj ≤0.05) and illustrated using GraphPad Prism (v9.4.1).

#### ASGARD analysis

To identify drugs capable of reprogramming NAT cells toward a healthy-like profile, the A Single-cell Guided Pipeline to Aid Repurposing of Drugs (ASGARD) package (v1.0.0) was employed. Tumor samples were excluded using the subset function. A reference library was created using the PrepareReference function. Differential gene expression profiles for every cell type were identified using the limma package (v3.52.4). NAT samples were set as cases, and healthy samples as controls. For drug identification, the tissue-specific drug reference “large-intestine” was loaded, and mono-drug repurposing for every cell type was performed. Finally, drugs were identified using the DrugScore function and exported as an Excel file. The file was then sorted by *p* ≤ 0.05 and ordered based on the value of the drug therapeutic score.

#### Marker identification

For the scRNA-seq analysis, epithelial cells were isolated from the whole dataset using the subset function (Seurat). Differentially expressed genes were identified for NAT vs. healthy, tumor vs. healthy, and tumor vs. NAT using the FindMarkers function of the Seurat package (min.pct = 0.1, logfc.threshold = 0.25). Results were filtered based on log_2_FC ≥ 0.7 and padj ≤0.05 for upregulated genes and log_2_FC ≤ −0.7 and padj ≤0.05 for downregulated genes.

For the spatial transcriptomics dataset, markers were applied based on the previously characterized cell types.

The identified genes from both scRNA-seq and spatial transcriptomics datasets were compared using the bioVenn website (https://www.biovenn.nl/index.php) to identify overlapping genes. Genes were then ordered based on the log_2_FC of NAT/tumor vs. healthy in the scRNA-seq dataset. Then, the difference between the log_2_FC of NAT vs. healthy and tumor vs. healthy was calculated, and the genes were reordered based on this difference. Genes with a difference ≥0.5 and up/downregulation in both NAT and tumor compared to healthy were selected as potential gradient markers. In a next step, genes with log_2_FC ≥ 0.7 (upregulated) or log_2_FC ≤ −0.7 (downregulated) for NAT vs. healthy were selected. The expression level of these potential markers was analyzed in the bulk RNA-seq, scRNA-seq, serial biopsy RNA-seq, and spatial transcriptomics datasets. For scRNA-seq data, the expression level of the potential markers was plotted as boxplots using GraphPad Prism (v9.4.1).

#### Quantitative real-time PCR (qRT-PCR)

RNA was isolated using TRIzol (Invitrogen) and reverse transcribed using Moloney Murine Leukemia Virus reverse transcriptase (New England Biolabs) with random primers. Gene expression analysis was performed using quantitative real-time PCR with SYBR Green I (Roche Diagnostics). All expression values were normalized to 18S rRNA levels. Following primers were used in this study: *RNA18SN1* forward 5′- ACTGCCATTAAGGGTGTGGG-3′, reverse 5′- CAGGTCTTCACGGAGCTTGT-3’; *RPS19* forward 5′-GCCTGGAGTTACTGTAAAAGACG-3′, reverse 5′-CCCATAGATCTTGGTCATGGAGC-3’; *RPL30* forward 5′-TACGTCCTGGGGTACAAGCA-3′, reverse 5′-AAAGCTGGGCAGTTGTTAGC-3’; *RPS18* forward 5′-TGTGGTGTTGAGGAAAGCA-3′, reverse 5′-CTTCAGTCGCTCCAGGTCTT-3’; *RPS27A* forward 5′-CTGGAAGATGGACGTACTTTGTC-3′, reverse 5′-CGACGAAGGCGACTAATTTTGC-3’.

### Quantification and statistical analysis

Statistical analysis was performed using GraphPad Prism 10 software. Data were presented as means and SEM is indicated by the error bars. For the comparison of two conditions, Student’s *t* test was performed. One-way ANOVA was used for the comparisons of more than two groups. The significance of the comparison was illustrated using stars (∗p˂0.05, ∗∗p˂0.01, ∗∗∗p˂0.001, ∗∗∗∗p˂0.0001).

## Supplemental information

Document S1. Figures S1–S7

Table S1. Cell type numbers

Table S2. GSEA_sc_C5_all cells

Table S3. GSEA_sc_NAT_vs._HC_C5_all_Validation_data

Table S4. DEGs_All cell types

Table S5. DEGs_B cells

Table S6. DEGs_CD8 T cells

Table S7. DEGs_Endothelial cells

Table S8. DEGs_Epithelial cells

Table S9. DEGs_Macrophages

Table S10. DEGs_Mast cells

Table S11. DEGs_Myeloid cells

Table S12. DEGs_NK cells

Table S13. DEGs_Plasma cells

Table S14. DEGs_Stromal cells

Table S15. DEGs_Th2 cells

Table S16. DEGs_Tregs

Table S17. DEGs_Neutrophils

Table S18. GSEA_sc_C5_B cells

Table S19. GSEA_sc_C5_CD8 T cells

Table S20. GSEA_sc_C5_Endothelial cells

Table S21. GSEA_sc_C5_Epithelial cells

Table S22. GSEA_sc_C5_Macrophages

Table S23. GSEA_sc_C5_Mast cells

Table S24. GSEA_sc_C5_Myeloid cells

Table S25. GSEA_sc_C5_NK cells

Table S26. GSEA_sc_C5_Plasma cells

Table S27. GSEA_sc_C5_Stromal cells

Table S28. GSEA_sc_C5_TH2 cells

Table S29. GSEA_sc_C5_Tregs

Table S30. GSEA_sc_C5_Neutrophils

Table S31. GSEA_sc_C5_Epithelial_Valitation_Data

Table S32. ASGARD_Drugs_NATvs.healthy

Table S33. DEGs_Spatial_vs._scRNAseq

Table S34. DEGs_Epithelial_cells_analysis_vs._validation_dataset
