# Supplementary material for: Challenging the control through a single-cell perspective on normal adjacent tissue in colorectal cancer
Source: iScience. 2026 Apr 9;29(6):115671. doi: 10.1016/j.isci.2026.115671 (PMC13276536; doi:10.1016/j.isci.2026.115671)
Supplement: Document S1. Figures S1–S7 [file mmc1.pdf]

iScience, Volume *29*

Supplemental information

Challenging the control through a single-cell perspective on normal adjacent tissue in colorectal cancer

Patricia Raude, Onur Mert Batmaz, Subhiksha Meenakshi Sundaram, Christina Parpoulas, Xiaodong Wang, Joana Aggrey Fynn, Jana Koch, Dina Mönch, Thomas Mürdter, Marc H. Dahlke, Dominik Saul, and Robyn Laura Kosinsky

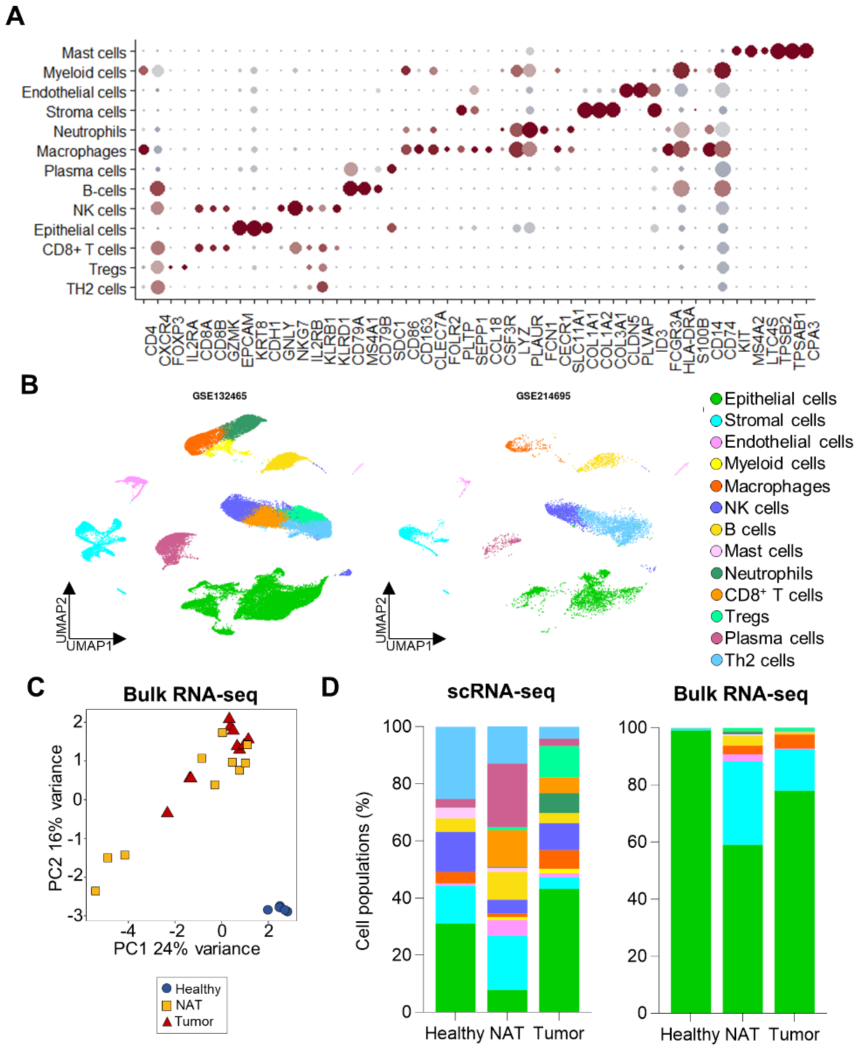

Supplementary Figure S1: Analysis of cell type composition across different datasets. (A) Expression levels of marker genes used for cell type annotation in single-cell RNA sequencing data (GSE132465 and GSE214695). (B) UMAP visualizations of the individual datasets GSE132465 and GSE214695 following cell type annotation, highlighting the distribution of identified cell types. (C) PCA plot of bulk RNA-seq data (GSE95132), showing the separation of healthy, NAT, and tumor samples. (D) Stacked bar diagrams illustrating the relative percentage of each cell type present in healthy, NAT, and tumor samples. The first diagram displays cell type proportions derived from scRNA-seq data (GSE132465 and GSE214695), while the second diagram represents deconvoluted bulk RNA-seq data (GSE95132) using the MuSiC tool [28,29].

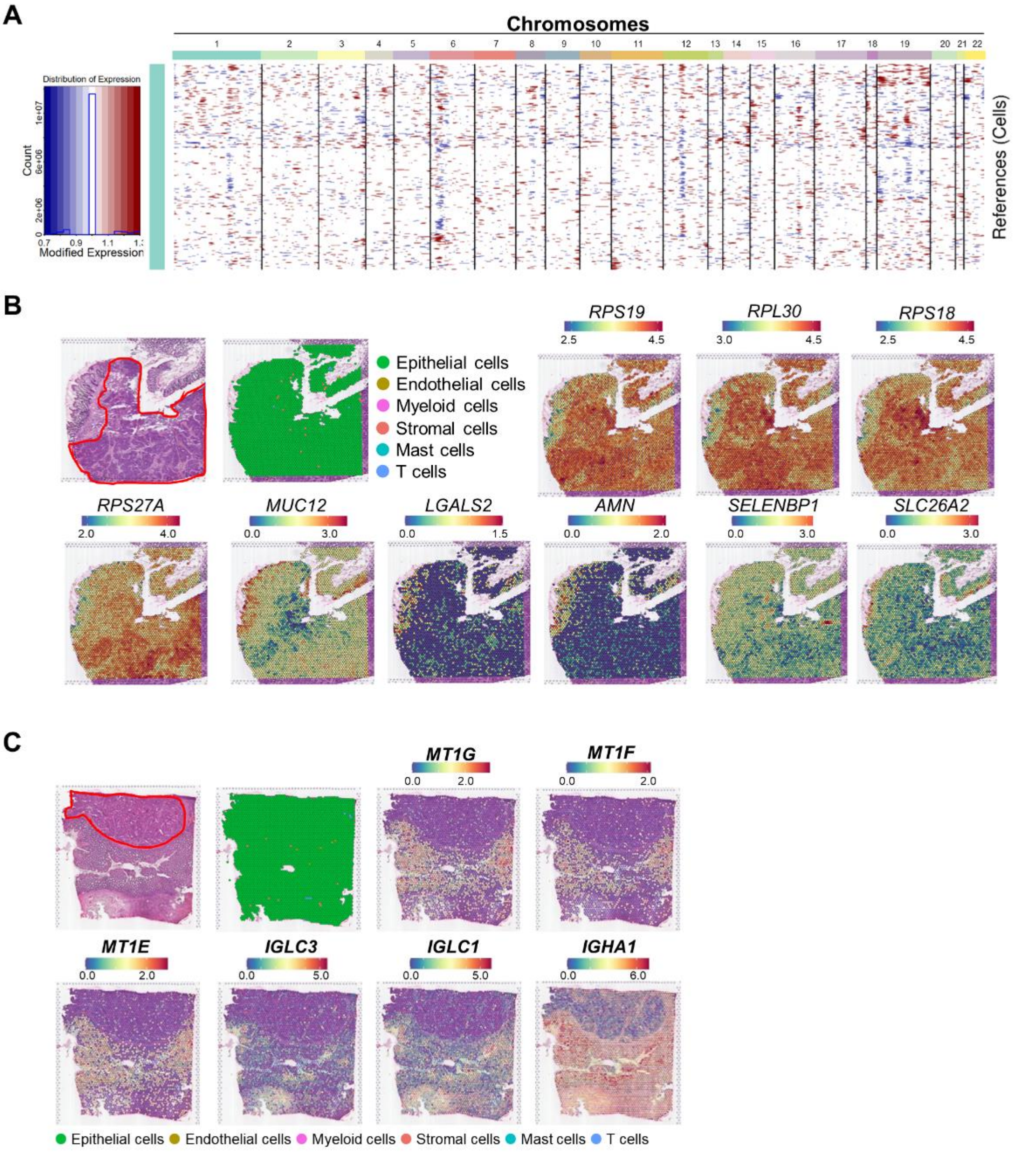

Supplementary Figure S2: Analysis of copy number variations and spatial transcriptomics results. (A) Healthy epithelial cell reference that was used for the comparison of CNVs between NAT and tumor cells. CNVs were identified using the inferCNV package. (B) Representative spatial transcriptomics analysis [13] from an additional colorectal tumor, highlighting detected cell types alongside H&E staining with marked tumor regions (red). Expression levels of nine markers are visualized, with blue indicating low expression and red indicating high expression. (C) Gene expression in spatial transcriptomic data of respresentative differentially expressed genes between tumor and NAT, identified in scRNA-seq data.

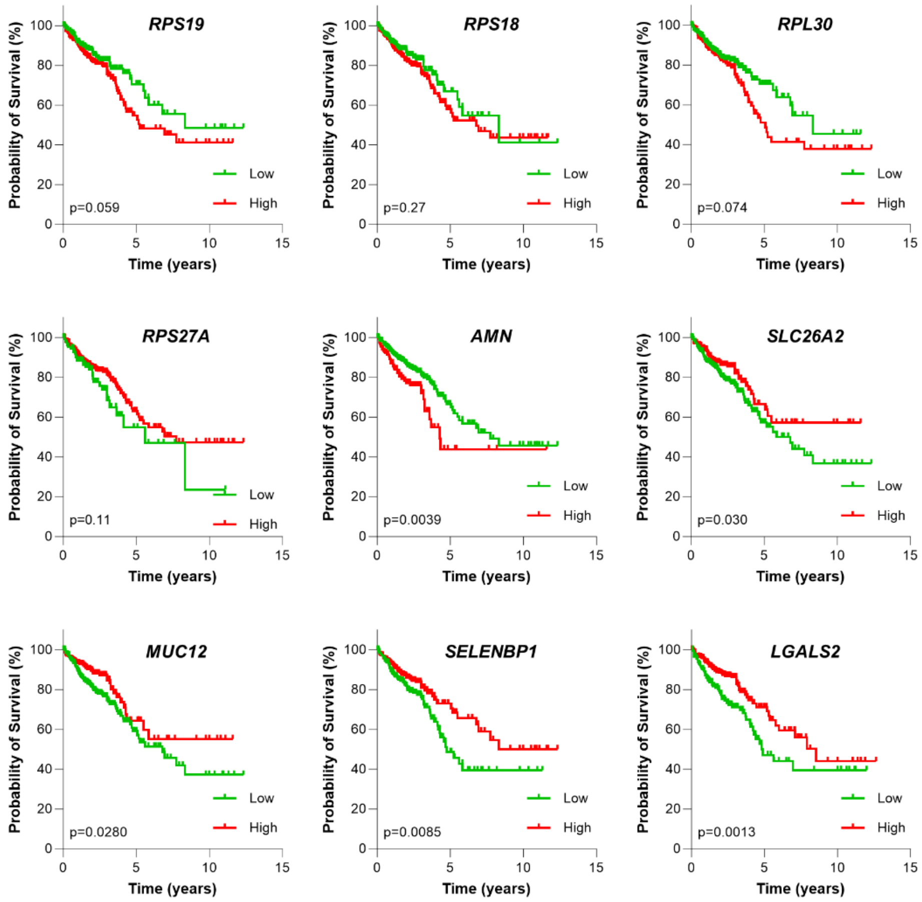

Supplementary Figure S3: Association of marker gene expression with the survival of CRC patients. Kaplan-Meier survival curves for CRC patients stratified by the expression levels of *RPS19*, *RPS18*, *RPL30*, *RPS27A*, *AMN*, *SLC26A2*, *MUC12*, *SELENBP1*, and *LGALS2* as determined from publicly available TCGA data on the Human Protein Atlas website. The "best expression cut-off" suggested by the website was used to categorize patients into high and low expression groups (*RPS19:* 328.42 FPKM, high n = 319, low n = 278; *RPS18:* 1085.9 FPKM, high n = 331, low n = 266; *RPL30:* 206.17 FPKM, high n = 214, low n = 215; *RPS27A:* 150.85 FPKM, high n = 466, low n = 131; *AMN:* 14.64 FPKM, high n = 165, low n = 432; *SLC26A2:* 6.29 FPKM, high n = 280, low n = 317; *MUC12:* 9.34 FPKM, high n = 247, low n = 248; *SELENBP1:* 50.47 FPKM, high n = 342, low n = 255; and *LGALS2:* 2.51 FPKM high n = 393, low n = 204).

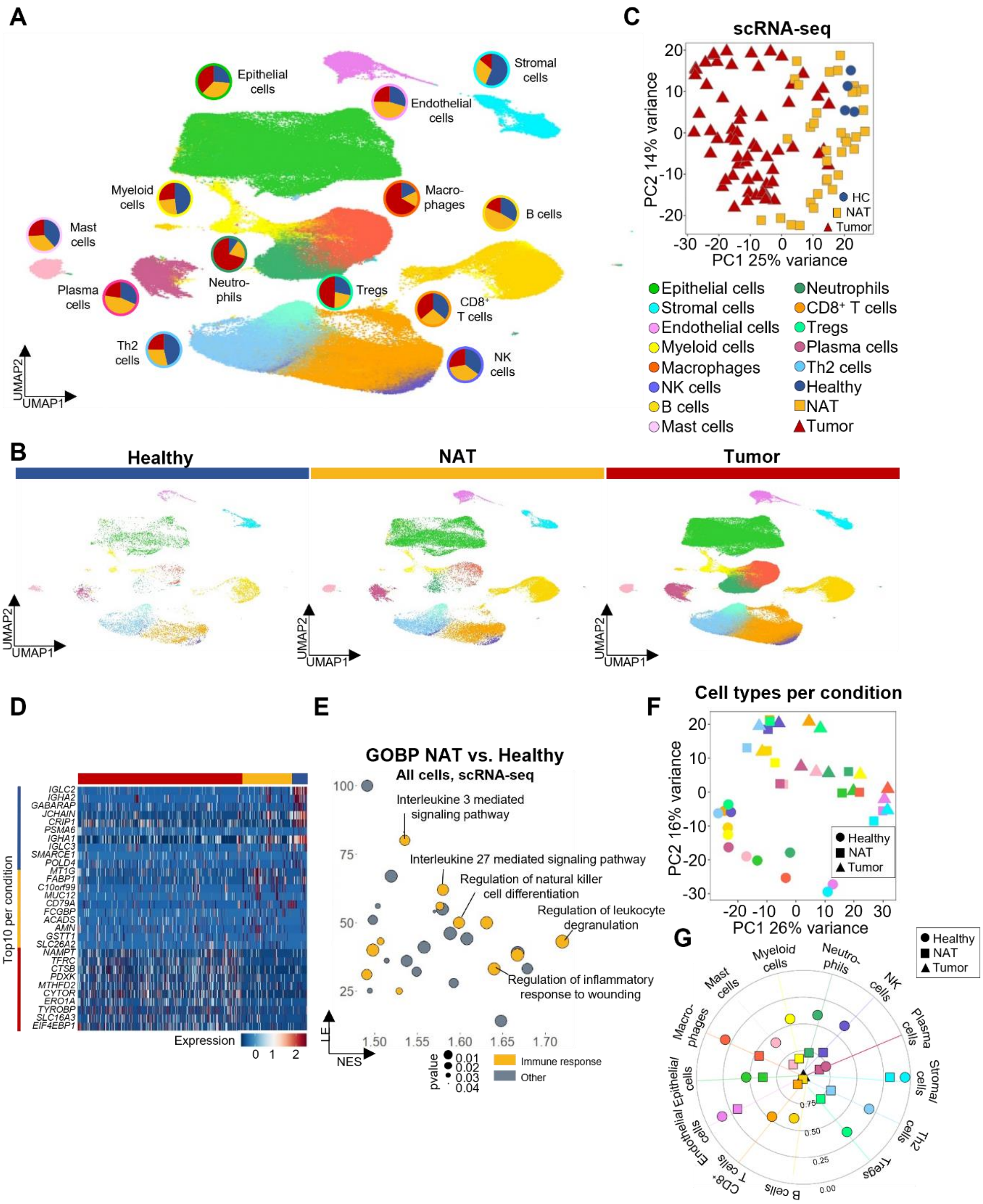
A
Epithelial cells
Stromal cells
Endothelial cells
Myeloid cells
Macro-phages
B cells
Mast cells
Neutro-phils
Plasma cells
Tregs
CD8⁺ T cells
Th2 cells
NK cells
UMAP2
UMAP1
B
Healthy
NAT
Tumor
C
scRNA-seq
PC2 14% variance
PC1 25% variance
HC
NAT
Tumor
Epithelial cells
Stromal cells
Endothelial cells
Myeloid cells
Macrophages
NK cells
B cells
Mast cells
Neutrophils
CD8⁺ T cells
Tregs
Plasma cells
Th2 cells
Healthy
NAT
Tumor
D
Top10 per condition
IGLC2
IGHA2
GABARAP
JCHAIN
CRIP1
PSMA6
IGHA1
IGLC3
SMARCE1
POLD4
MT1G
FABP1
C10orf99
MUC12
CD79A
FCGBP
ACADS
AMN
GSTT1
SLC26A2
NAMPT
TFRC
CTSB
PDXK
MTHFD2
CYTOR
ERO1A
TYROBP
SLC16A3
EIF4EBP1
Expression
E
GOBP NAT vs. Healthy
All cells, scRNA-seq
Interleukine 3 mediated signaling pathway
Interleukine 27 mediated signaling pathway
Regulation of natural killer cell differentiation
Regulation of leukocyte degranulation
Regulation of inflammatory response to wounding
LE
NES
pvalue
Immune response
Other
F
Cell types per condition
PC2 16% variance
PC1 26% variance
Healthy
NAT
Tumor
G
Healthy
NAT
Tumor
Myeloid cells
Neutro-phils
NK cells
Plasma cells
Stromal cells
Th2 cells
Tregs
B cells
CD8⁺ T cells
Endothelial cells
Epithelial cells
Macro-phages
Mast cells

Supplementary Figure S4: Validation of scRNA-seq data in a second independent dataset. ScRNA-seq data of healthy colon material (GSE231993 [35]) was integrated and harmonized with a dataset containing NAT and colorectal tumors (GSE178341 [36]). This validation dataset comprised 4 healthy, 26 NAT, and 62 tumor samples. (A) UMAPs illustrating thirteen distinct cell types identified in the dataset. Pie charts indicate the proportion of cells originating from healthy (blue), NAT (orange), and tumor (red) tissue per cell type. (B) UMAPs segregated into healthy, NAT, and tumor samples, demonstrating differences in cell type composition. (C) PCA plot of pseudo-bulk scRNA-seq data depicting the similarities between samples based on expression patterns in healthy (●; n = 4), NAT (■; n = 26) and tumor (▲; n = 62). (D) Heatmap illustrating the top ten DEGs in healthy, NAT, and tumor cells ($log_2FC > 0.7$, padj < 0.05; blue: low expression, red: high expression). (E) Gene Ontology of Biological Processes (GOBP) in NAT compared to healthy tissue from all cells reveals that the key differences are linked to immune response-associated processes (yellow). NES: normalized enrichment score, LE: leading edge, dot size inversely proportional to the adjusted p-value. (F) PCA plot of pseudo-bulk scRNA-seq data illustrating the distinct expression patterns in healthy, NAT, and tumor cells for all cell types. NAT frequently appears in a state intermediate between healthy and tumor. (G) Based on PCA, distances from NAT and healthy cells to tumor cells were determined for each cell type. Values were normalized to the tumor condition and visualized in a circular plot. NAT consistently appears in an intermediate position between healthy and the central tumorous state.

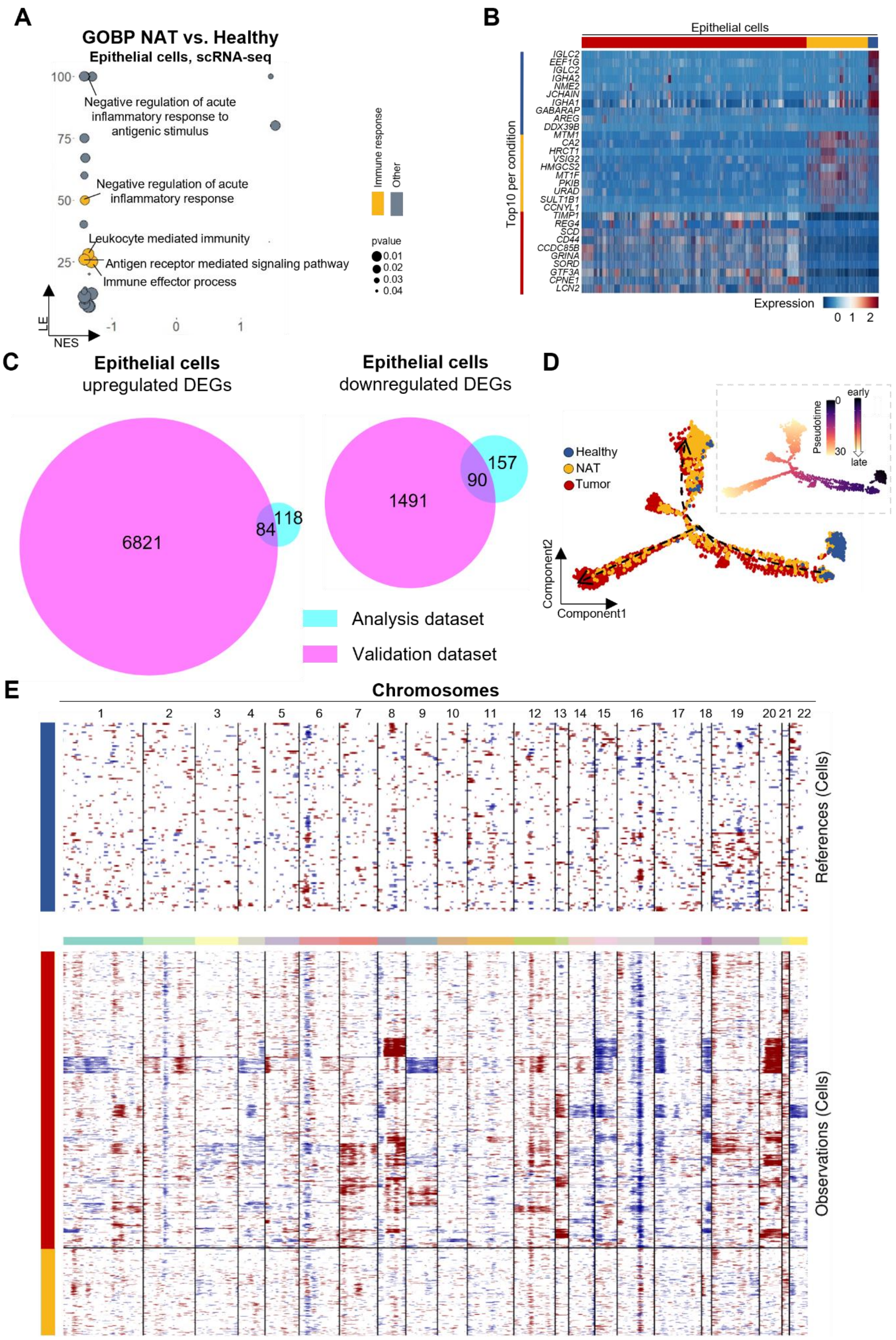
A
GOBP NAT vs. Healthy
Epithelial cells, scRNA-seq
Negative regulation of acute inflammatory response to antigenic stimulus
Negative regulation of acute inflammatory response
Leukocyte mediated immunity
Antigen receptor mediated signaling pathway
Immune effector process
Immune response
Other
pvalue
0.01
0.02
0.03
0.04
LE
NES
B
Epithelial cells
Top10 per condition
IGLC2
EEF1G
IGLC2
IGHA2
NME2
JCHAIN
IGHA1
GABARAP
AREG
DDX39B
MTM1
CA2
HRCT1
VSIG2
HMGCS2
MT1F
PKIB
URAD
SULT1B1
CCNYL1
TIMP1
REG4
SCD
CD44
CCDC85B
GRINA
SORD
GTF3A
CPNE1
LCN2
Expression
C
Epithelial cells
upregulated DEGs
6821
84
118
Epithelial cells
downregulated DEGs
1491
90
157
Analysis dataset
Validation dataset
D
Healthy
NAT
Tumor
Pseudotime
early
late
Component2
Component1
E
Chromosomes
References (Cells)
Observations (Cells)

Supplementary Figure S5: NAT-derived epithelial cell characterization in a scRNA-seq validation dataset. (A) Gene Ontology of Biological Processes (GOBP) in NAT versus healthy tissue, focusing exclusively on epithelial cells, reveals that the key differences are linked to immune response (yellow). NES: normalized enrichment score, LE: leading edge, dot size inversely proportional to the adjusted p-value. (B) Heatmap depicting the top 10 DEGs in healthy, NAT, and tumor epithelial cells ($log_2FC > 0.7$, padj < 0.05). (C) Venn diagram of DEGs in epithelial cells of the analysis dataset (blue) compared to the validation dataset (pink) ($log_2FC > 0.7$, padj < 0.05, Supplementary Table S34). (D) The developmental dynamics of epithelial cells were further evaluated using monocle2 [12], indicating early (purple) and late (yellow) fates (right image), with the developmental direction marked by an arrow. (E) Genome-wide determination of copy number variations using the inferCNV package referencing healthy epithelial cells (blue). Average signal across all CRC patients revealed common CNVs in NAT (yellow) and tumor cells (red).

A

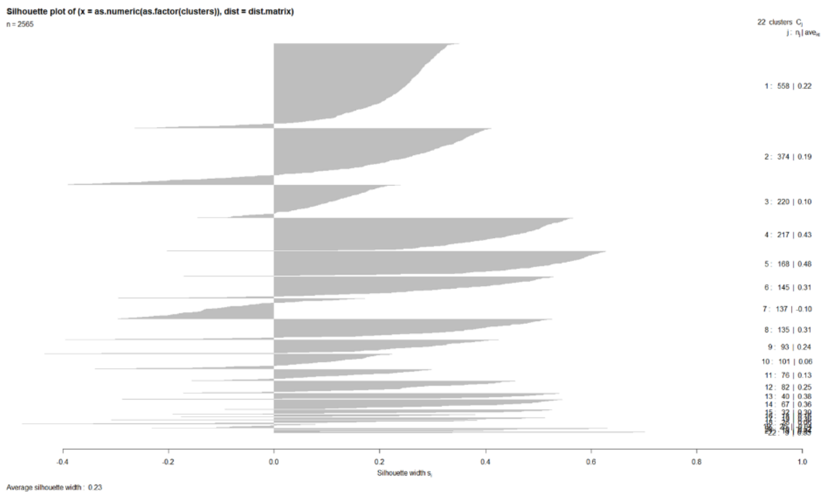

B

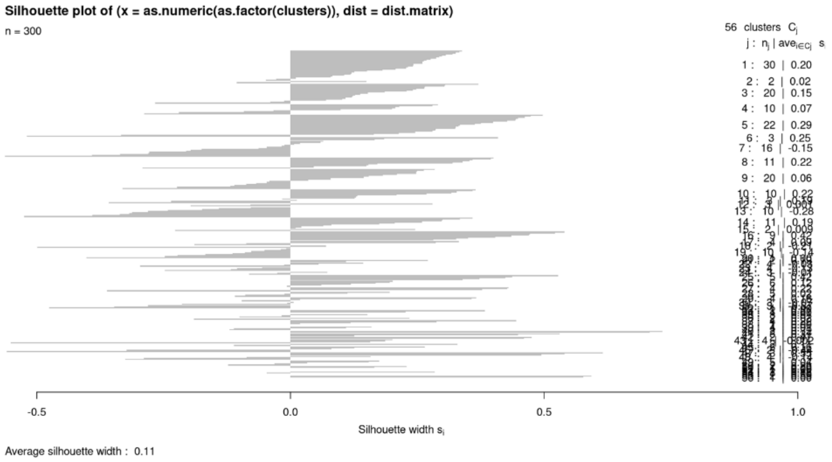

Supplementary Figure S6: Silhouette plot of the integrated and harmonized analysis scRNA-seq (A) and scRNA-seq validation (B) dataset. The average silhouette width across all cells was 0.23 (A) / 0.11 (B), reflecting a moderate separation of transcriptionally distinct cell populations after integration and batch correction using Harmony.

A

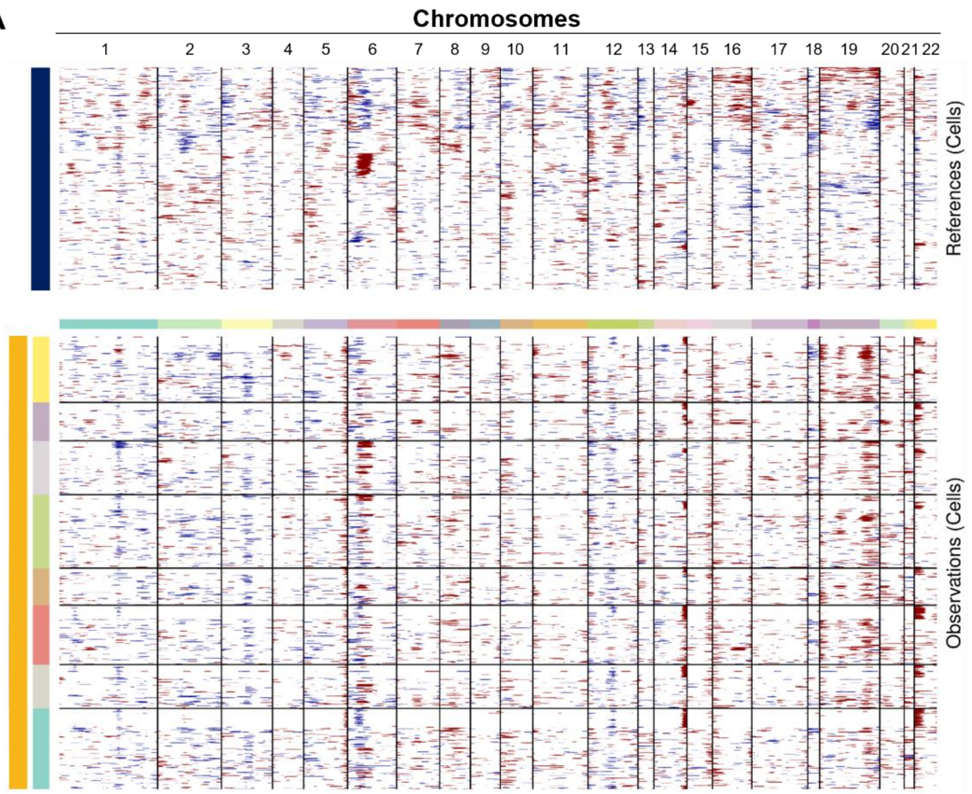

Chromosomes
1 2 3 4 5 6 7 8 9 10 11 12 13 14 15 16 17 18 19 20 21 22
References (Cells)
Observations (Cells)

B

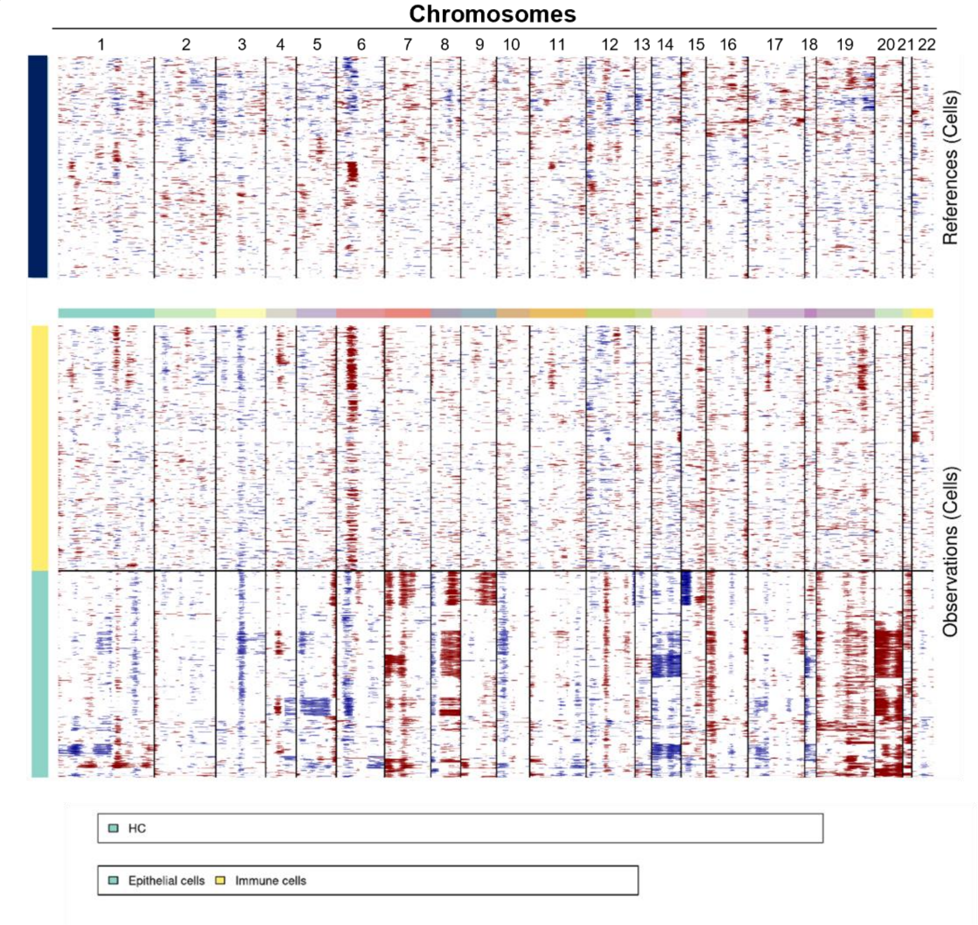

Chromosomes
1 2 3 4 5 6 7 8 9 10 11 12 13 14 15 16 17 18 19 20 21 22
References (Cells)
Observations (Cells)
HC
Epithelial cells
Immune cells

Supplementary Figure S7: InferCNV analysis of intra-sample heterogeneity among NAT cells (A) and Epithelial cells compared to immune cells in the tumor subset (B). comparisons using th analysis dataset. (A) **Intra-sample heterogeneity among NAT cells.** The eight NAT samples (SMC01, -02, -04, -05, -07, -08, -09, -10; bottom panel) display comparable CNVs when compared to healthy control epithelial cells (HC; top panel). (B) Epithelial cells compared to immune cells in the tumor subset. CNVs were compared between immune (T cells, B cells and myeloid cells) and epithelial cells (bottom panel). Healthy control cells were used as a reference (HC; top panel).
